# Breed-specific microbiomes drive differential responses to 3-nitrooxypropanol and *Acacia mearnsii* in dairy cows

**DOI:** 10.3168/jdsc.2025-0897

**Published:** 2026-02-19

**Authors:** M.Z. Islam, D.W. Pitta, M. Niu

**Affiliations:** 1 Animal Nutrition, Institute of Agricultural Sciences, Department of Environmental Systems Science, ETH Zürich, Zürich 8092, Switzerland; 2 Department of Clinical Studies, University of Pennsylvania, School of Veterinary Medicine, New Bolton Center, Kennett Square, PA 19348

## Abstract

Methane (CH_4_) inhibitor 3-nitrooxypropanol (3-NOP) shows variable efficacy in Brown Swiss (BS) and Holstein (HF) cows, but the underlying mechanisms remain unclear. Rumination bolus, a proxy for rumen microbiota, combined with exhalomics (analysis of exhaled volatiles), offered a noninvasive approach to differentiate breed responses to CH_4_ mitigation strategies. We investigated whether differences in rumination bolus microbiota and exhaled VFA (eVFA) between BS and HF cows contribute to their responses to 3-NOP. Sixteen multiparous cows (8 BS, 8 HF) were studied in a replicated 4 × 4 Latin square design with a 2 × 2 factorial arrangement of 3-NOP (0 or 60 mg/kg of DM) and *Acacia mearnsii* tannin extract (TAN; 0% or 3% of DM). Rumination bolus samples collected 6 h postfeeding during the fourth period of the experiment were sequenced for full-length 16S rRNA sequences (PacBio Revio platform) to assess bacterial diversity, while exhaled samples from all 4 periods were analyzed for VFA using secondary electrospray ionization-MS. Microbial community shifts were analyzed using Bray–Curtis, unweighted, and weighted unique fraction metric (UniFrac) distances. Spearman correlations were performed between genus-level information and eVFA, CH_4_, hydrogen (H_2_), carbon dioxide (CO_2_), DMI, and milk yield between breeds. Supplementation of 3-NOP induced a modest but consistent shift in microbial composition, whereas TAN effects were minimal and inconsistent. Breed-specific differences were evident: BS harbored more *Prevotella* and *Rikenellaceae*, and HF were enriched in *Succinivibrionaceae* and *Acetitomaculum*. Under 3-NOP, HF genera aligned more strongly with propionate-producing pathways and showed stronger negative correlations with CH_4_, and BS genera remained more associated with acetate and butyrate proportions. Correlations with feed intake and milk yield were generally weak and inconsistent across genera. Overall, our results indicate that HF cows are more responsive to 3-NOP, likely redirecting spared H_2_ toward propionate-producing bacteria, and BS are more resilient to H_2_ fluxes and consequently less responsive to 3-NOP. Findings from this pilot study highlight the importance of host-microbiome interactions in evaluating and implementing enteric CH_4_ mitigation strategies in dairy cattle.

## Highlights

• Brown Swiss and Holsteins do not respond the same to 3-NOP.
• Brown Swiss retained acetate- and butyrate-linked microbes with 3-NOP.
• Spared hydrogen was redirected to propionate only in Holsteins, not Brown Swiss.
• Rumination bolus and breath VFA revealed breed-specific fermentation routes.

## Graphical Abstract

**Figure:**
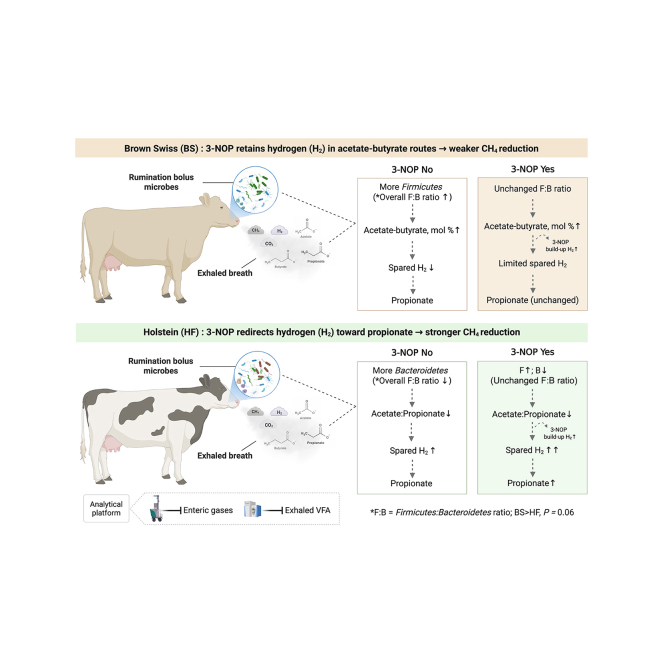
**Summary:** We asked why the methane inhibitor 3-nitrooxypropanol (3-NOP) works differently across dairy breeds. Sixteen multiparous cows (8 Brown Swiss, 8 Holstein) were studied in a replicated Latin square with 3-NOP (0 or 60 mg/kg of dry matter [DM]) and *Acacia mearnsii* tannin extract (TAN; 0% or 3% of DM). Rumination-bolus samples were sequenced for 16S ribosomal ribonucleic acid to characterize microbial communities, and exhaled volatile fatty acids (VFA) were analyzed by mass spectrometry. Supplementation with 3-NOP induced modest but consistent microbial shifts, whereas TAN had minimal effects. Breed-specific responses were evident: Holsteins redirected hydrogen toward propionate-associated genera and showed stronger negative correlations with methane, whereas Brown Swiss maintained acetate- and butyrate-linked associations and responded less to 3-NOP. Correlations between microbial genera and intake or milk yield were weak. These findings indicate that host-microbiome interactions underlie divergent responses to inhibitors and highlight the need to consider breed-specific microbial ecology when designing methane-mitigation strategies for dairy production. Created in BioRender: https://BioRender.com/8qytezx.

Methane (CH_4_) from enteric fermentation is a potent GHG and represents an energy loss to the cow (Hristov et al., 2013). Dairy breeds differ in rumen microbial communities and fermentation profiles (Paz et al., 2016; Gonzalez-Recio et al., 2018), traits that are mechanistically linked to variation in enteric CH_4_ emissions. Recent multi-omics evidence further shows that interbreed differences in CH_4_ yield are explained by distinct microbial hydrogen (H_2_) and reductant disposal pathways (Li et al., 2024), whereby low-emitting breeds redirect reductant toward propionate and AA synthesis, and high-emitting breeds favor H_2_ production and methanogenesis. Within this context, recent studies indicate that Brown Swiss (**BS**) and Holstein (**HF**) cows can show divergent responses to 3-nitrooxypropanol (**3-NOP**), despite receiving the same dose (Ma et al., 2024; Islam et al., 2025), suggesting that breed-specific rumen ecology may condition mitigation efficacy. Among dietary CH_4_ mitigation strategies, 3-NOP is one of the most effective and consistent inhibitors for dairy cows (predominantly in HF), demonstrating ∼30% reductions (Kebreab et al., 2023) and no adverse effect on the lactation performance of dairy cows (Martins et al., 2025). Plant secondary compounds such as *Acacia mearnsii* tannin extracts (**TAN**) have also been explored, and evidence for synergy with 3-NOP is limited (Islam et al., 2025). As highlighted by Ungerfeld (2020), methanogenesis is the main H_2_ sink in the rumen, and variation in redirecting H_2_ to alternative sinks (e.g., propionate, reductive acetogenesis) may explain breed- or diet-dependent inhibitor efficacy. Studies in cattle and sheep comparing high- and low-CH_4_ phenotypes show that host-microbiome interactions, microbial pathways of H_2_ allocation, and associated VFA stoichiometry strongly shape CH_4_ yield (Kaplan-Shabtai et al., 2021; Stepanchenko et al., 2023). Even subtle shifts in acetate, propionate, and butyrate pathways can influence H_2_ redirection from methanogenesis. Methanogen diversity, H_2_ dynamics, and host genetics further contribute to variable outcomes (Pitta et al., 2022a), yet little is known about how dairy breeds differ in microbial H_2_ disposal strategies. This represents a key knowledge gap because dairy production relies on multiple breeds that may not respond uniformly to mitigation strategies. Building on our recent observation of a breed × 3-NOP interaction in CH_4_ emissions (Islam et al., 2025), the objective of this study was to determine whether microbial composition contributes to this discrepancy and to evaluate associations between microbial diversity and exhaled VFA (**eVFA**) as indicators of H_2_ partitioning during CH_4_ inhibition. We hypothesize that the rumen microbiota and their metabolites differ between BS and HF cows, and that these breed-specific features underlie their differential responses to 3-NOP.

The animal experimental design and procedures were described in detail by Islam et al. (2025). In brief, 16 multiparous lactating cows (8 BS and 8 HF) were arranged in a split-plot design, with breed as the main plot and a replicated 4 × 4 Latin square as the subplot. These cows had been maintained under identical farm management conditions since their first lactation, and all cows were managed identically throughout the trial. The factorial arrangement consisted of 2 levels of 3-NOP (0 or 60 mg/kg of DM, Bovaer10, DSM Nutritional Products, Basel, Switzerland) and 2 levels of TAN (0% or 3% of DM; Weibull Black, TENAC, Brazil). Each 24-d period included 17 d of adaptation in freestall housing, 4 d in tiestalls, and 3 d of sampling. At the start of the trial, HF cows averaged (mean ± SD) 134 ± 31 DIM, 35.8 ± 6.04 kg/d of milk yield, and 710 ± 68.0 kg BW, whereas BS averaged 161 ± 40 DIM, 27.0 ± 6.24 kg/d, and 710 ± 63.0 kg BW. Diets were offered ad libitum (110% of previous intake) twice daily at 0800 and 1800 h. The 4 treatments were as follows: basal control diet (**CON**), CON + 3-NOP, CON + TAN, and CON + 3-NOP + TAN.

For bacterial diversity analysis, we chose to use the rumination bolus (regurgitated digesta), given the limitations of using either a cannula-derived or stomach-tube-derived rumen sample. Recent studies have shown that rumination bolus samples collected 6 h postfeeding are a good proxy for ruminal digesta (Indugu et al., 2021; Castaneda et al., 2025). One cow was excluded from sampling due to health issues unrelated to the treatments. Cows were continuously monitored, and once rumination began, a portion of the regurgitated bolus was manually retrieved ∼6 h postfeeding (only from the fourth period of the experiment). Samples were placed into tubes prefilled with DNA/RNA shield (Zymo Research, CA) and stored at −20°C until analysis. Genomic DNA was extracted, and full-length 16S rRNA genes were PCR-amplified with universal primers (AGRGTTYGATYMTGGCTCAG / RGYTACCTTGTTACGACTT). Sequencing was performed on the PacBio Revio platform (HiFi reads, Pacific Biosciences, Menlo Park, CA), yielding ∼30,000 clean reads per sample. Reads were denoised with DADA2, low-abundance amplicon sequence variants (**ASV**, <5 reads across all samples) were removed, and taxonomy was assigned in QIIME2 (Bolyen et al., 2019; https://qiime2.org) using the SILVA 138.1 reference database (https://www.arb-silva.de) with the classify-sklearn algorithm.

Enteric emissions of CH_4_, H_2_, and CO_2_ were measured across 8 times per period using the GreenFeed system (C-Lock Inc., Rapid City, SD) as described by Islam et al. (2025). Simultaneously, exhaled breath (all exhaled volatiles) was collected at the same 8 time points into Tedlar bags (Thermogreen LB-2 septa, Merck, St. Louis, MO), and secondary electrospray ionization-MS was used to quantify eVFA following the method described by Islam et al. (2023, 2024). Profiles were expressed as molar proportions (**mol %**) of individual eVFA relative to the total ion count. For consistency with rumination bolus sampling, only the measurements obtained from 6 h postfeeding were used for the downstream correlation analysis. Given the different data structures, 2 analytical frameworks were applied. Microbiome analyses were based on a single bolus sample collected once per cow during period 4 (6 h postfeeding) and were therefore analyzed using a linear model. In contrast, eVFA data were collected repeatedly across all 4 experimental periods and were analyzed using linear mixed-effects models as described in Islam et al. (2025).

To focus on dominant taxa, genera with mean relative abundance <0.1% were excluded. *Firmicutes:Bacteroidetes* (**F:B**) ratios, calculated from relative abundances, were visualized as bar plots (arithmetic means ± SE) and compared by breed and breed × 3-NOP interaction. Microbial community structure was assessed using Bray–Curtis dissimilarity, unweighted unique fraction metric (**UniFrac**), and weighted UniFrac (computed from ASV counts within a *phyloseq* [McMurdie and Holmes, 2014] object). Distance matrices were ordinated by principal coordinate analysis, with samples colored by breed and shaped by 3-NOP. Group differences were tested using permutational ANOVA (**PERMANOVA**; adonis2, vegan v2.6-4; Oksanen et al., 2005) with a model that included breed × 3-NOP and breed × TAN interactions; because each cow contributed a single microbiome sample, permutations were performed at the cow level (999 permutations). Homogeneity of dispersion was verified using *betadisper* function from the vegan R package. Statistical significance was declared at *P* ≤ 0.05, and 0.05 < *P* ≤ 0.10 was considered a tendency. To explore associations between microbial composition and host traits, genus-level relative abundances were paired with period 4, 6 h postfeeding measurements of eVFA proportions, enteric emissions, and daily DMI and milk yield. Within each breed and stratified by 3-NOP (averaged over TAN), Spearman's rank correlations were computed and visualized as heatmaps, with significance assessed after false discovery rate (**FDR**) correction. All statistical procedures were performed in the R statistical language (R Core Team, 2025; version 4.5.1).

At the community level, based on the Bray–Curtis dissimilarity index (Figure 1A), we observed no breed effect, but a significant effect of 3-NOP (PERMANOVA, *P* = 0.01; R^2^ = 8.31%), and a nonsignificant numerical shift for TAN (*P* = 0.13; R^2^ = 7.46%). The results suggest that although substantial variation exists among samples within each breed, 3-NOP reduces this variation, indicating a more consistent shift in bacterial communities across both breeds. Supplementation with TAN appeared to elicit a similar effect, but not as substantial as that of 3-NOP. To further assess whether these changes reflected differences in overall community structure or in specific microbial taxa, we analyzed both unweighted and weighted UniFrac distance matrixes (Figure 1, Figure 1). No differences were detected with the weighted UniFrac analysis, whereas the unweighted UniFrac analysis showed patterns similar to Bray–Curtis; however, the effect of 3-NOP remained nonsignificant (*P* = 0.17).

**Figure 1:**
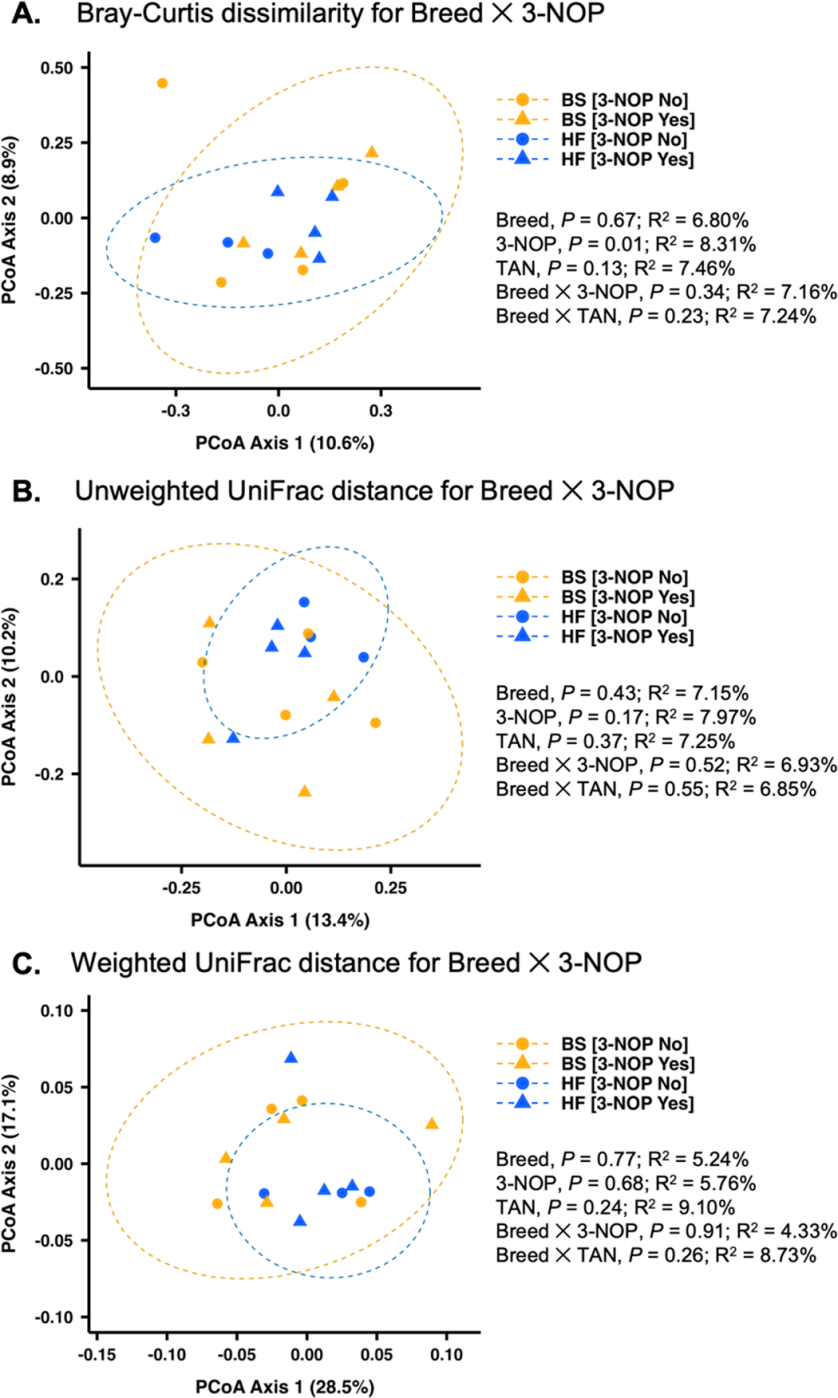
Principal coordinate analysis (PCoA) of bacterial ASV in rumination bolus of BS and Holstein (HF) cows, based on Bray–Curtis dissimilarity (A), unweighted UniFrac (B), and weighted UniFrac distance (C). The plot shows the effect of breed × 3-NOP, averaged over TAN supplementation. Colors indicate breed (orange = BS, blue = HF), and shapes indicate 3-NOP (circle = no, triangle = yes). Ellipses represent 95% CI for each breed. Differences among groups were tested using PERMANOVA, with R^2^ and *P-*values displayed in each panel. Dietary treatments were: CON = basal diet; 3-NOP (3-nitrooxypropanol); TAN (*Acacia mearnsii*); and 3-NOP + TAN. Factor-level definitions: [3-NOP No] = CON + TAN; [3-NOP Yes] = 3-NOP + 3-NOP+TAN.

At the phylum level, *Firmicutes* and *Bacteroidetes* dominated the rumen microbiota, accounting for up to 90% of the relative bacterial abundance. *Firmicutes* averaged 54% of the total community relative abundance and were similar between breeds, whereas *Bacteroidetes* represented ∼33% in BS and ∼36% in HF cows. Other phyla with >1% relative abundance included *Proteobacteria*, *Fibrobacteres*, *Spirochaetes*, *Actinobacteria*, *Planctomycetota*, and *Verrucomicrobia*, all of which were numerically higher in BS than in HF cows (data not shown). To further evaluate microbial community structure, we computed the F:B ratio across breeds and inhibitors as an indicator of gram-positive versus gram-negative populations. The F:B ratio tended to be higher in BS (1.68) than in HF cows (1.51; *P* = 0.06; Figure 2F). This is noteworthy because a greater relative abundance of *Firmicutes* is often linked to higher acetate production, whereas *Bacteroidetes* are typically associated with propionate production. Accordingly, we compared the mol % of acetate, propionate, butyrate, and the acetate:propionate (**A:P**) ratio between breeds (Figure 2A–D). Consistent with the similar abundance of *Firmicutes*, acetate proportions were also comparable between breeds. In contrast, the relatively higher abundance of *Bacteroidetes* in HF coincided with greater propionate proportions compared with BS. Finally, the higher A:P ratio in BS compared with HF is in line with their relatively greater F:B ratio, suggesting possible microbial contributions to eVFA partitioning.

**Figure 2:**
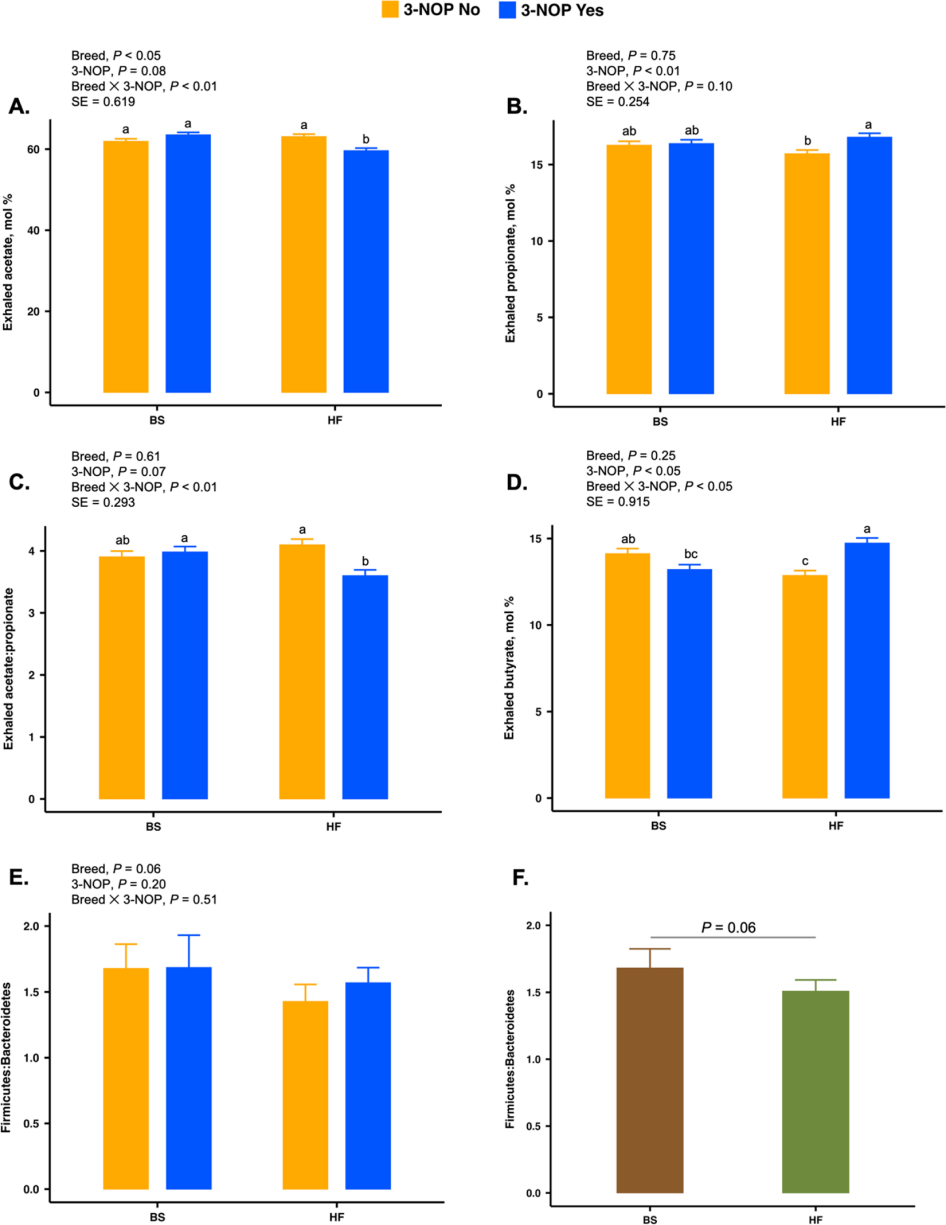
Interactions of 3-NOP and dairy cattle breed BS and Holstein Friesian (HF) on mol % of exhaled VFA, acetate (A), propionate (B), acetate-to-propionate ratio (C), and butyrate (D) measured via secondary electrospray ionization-MS. Data are presented as LSM ± SE with statistical inferences from a linear mixed model. Rumination bolus F:B ratio (E) and overall breed effect (F) are presented as arithmetic means ± SE, with statistical inferences for the F:B ratio derived from a linear model (data only from the fourth period of the experiment). Dietary treatments were as follows: CON = basal diet; 3-NOP (3-nitrooxypropanol); TAN (*Acacia mearnsii*); and 3-NOP + TAN. Factor-level definitions: [3-NOP No] is the LSM of CON + TAN diet; [3-NOP Yes] is the LSM of 3-NOP + 3-NOP+TAN diet. (A) Exhaled acetate [BS 3-NOP No] = 61.9^a^, [BS 3-NOP Yes] = 63.6^a^, [HF 3-NOP No] = 63.1^a^, [HF 3-NOP Yes] = 59.6^b^. (B) Exhaled propionate [BS 3-NOP No] = 16.3^ab^, [BS 3-NOP Yes] = 16.4^ab^, [HF 3-NOP No] = 15.7^b^, [HF 3-NOP Yes] = 16.8^a^. (C) Exhaled acetate-to-propionate ratio [BS 3-NOP No] = 3.91^ab^, [BS 3-NOP Yes] = 3.98^a^, [HF 3-NOP No] = 4.10^a^, [HF 3-NOP Yes] = 3.60^b^. (D) Exhaled butyrate [BS 3-NOP No] = 14.1^ab^, [BS 3-NOP Yes] = 13.2^bc^, [HF 3-NOP No] = 12.9^c^, [HF 3-NOP Yes] = 14.7^a^. (E) F:B ratio [BS 3-NOP No] = 1.68, [BS 3-NOP Yes] = 1.69, [HF 3-NOP No] = 1.43, [HF 3-NOP Yes] = 1.57. Different letters within a panel denote significant differences (*P* ≤ 0.05).

Interactions between breed and 3-NOP supplementation revealed distinct patterns in microbial community composition. In BS cows, 3-NOP induced only subtle shifts, with marginal increases in both *Firmicutes* and *Bacteroidetes*, leaving the F:B ratio essentially unchanged (Figure 2E). In HF cows, *Firmicutes* increased slightly while *Bacteroidetes* decreased numerically, but again these shifts did not translate into a notable change in the F:B ratio (Figure 2E). Among the less abundant phyla, *Fibrobacteres*, *Proteobacteria*, and *Spirochaetes* decreased, and *Actinobacteria* increased with 3-NOP in BS, whereas the opposite numerical trends were observed in HF. As mentioned earlier, independent of 3-NOP, BS cows tended to have a higher F:B ratio than HF cows. With these microbial patterns, the lack of F:B ratio changes in BS coincided with no alterations in acetate or the A:P ratio, whereas the numerical reduction in *Bacteroidetes* in HF was accompanied by a lower A:P ratio (Figure 2C). Together, these findings support the hypothesis that bacterial composition and eVFA profiles are linked and that such associations are influenced by breed-specific host phenotypes.

To better interpret genus-level differences, we performed correlation analyses between the abundant taxa, eVFA mol %, enteric gases, DMI, and milk yield. In BS, only a few genera exhibited strong correlations, whereas HF showed broader and stronger associations, suggesting an association consistent with redirection of H_2_ toward propionate formation. For example, *Acetitomaculum* and *Succiniclasticum* correlated positively with propionate, *Prevotella* with acetate, and *Butyrivibrio* with butyrate. These patterns indicate that HF cows maintain wider microbial-trait networks than BS, reflecting potential breed-specific contributions of dominant bacteria to fermentation and production traits. These correlations decreased in magnitude under 3-NOP (Figure 3A–B). In contrast, HF cows retained clearer microbial-trait associations, although weaker in magnitude, with *Succinivibrionaceae_UCG-001* remaining positively correlated with propionate across treatments (Figure 3C–D). Other propionate-associated genera lost correlations under inhibition, and new associations emerged with *Prevotellaceae* and *Papillibacter*, indicating that alternative propionate routes became more prominent when methanogenesis was suppressed in HF cows. Rapid-fermenting taxa such as *Solobacterium*, *Succinivibrionaceae*, and other *Proteobacteria* also shifted their associations: weak positive links with acetate turned negative, and new positive correlations with propionate or butyrate appeared (Figure 3D). This suggests that H_2_ spared from methanogenesis was redirected into alternative fermentation pathways; however, these results are associative and require validation of such associations in future studies. Despite the small sample size, this pilot study provides new insight into breed-specific microbiota-metabolome interactions, showing that natural variation in microbial community shapes how cows respond to CH_4_ inhibitors as reflected in eVFA profiles.

**Figure 3:**
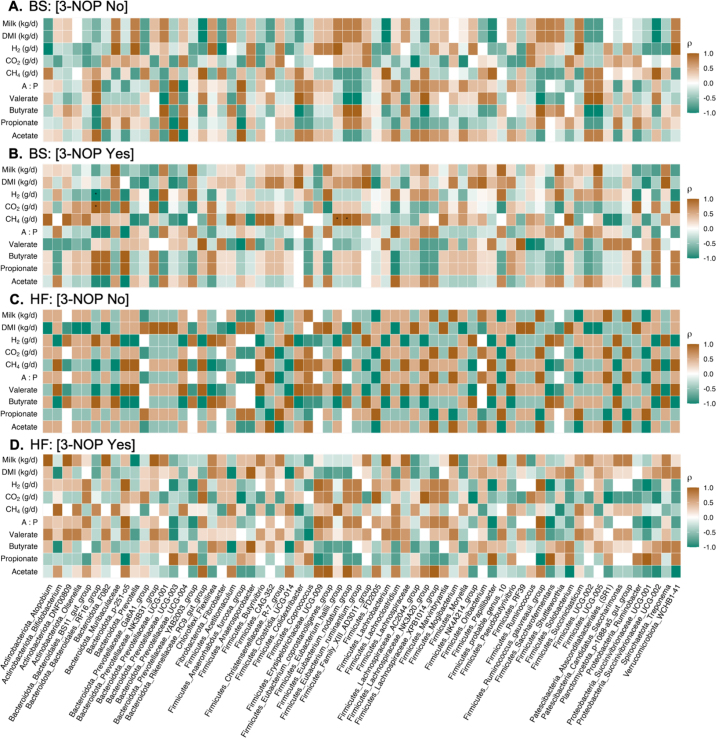
Spearman correlations (ρ) between rumination bolus microbial genera and fermentation or production traits in BS (A, B) and Holstein (HF; C, D) cows. Rows represent traits (exhaled VFA, enteric gases, DMI, and milk yield), and columns represent microbial taxa (taxa ordered alphabetically by phylum-genus structure across breeds to enable direct comparison). The color scale indicates the strength and direction of the correlation (brown = positive, green = negative), and asterisks (*) mark significant correlations within each condition (FDR; *P* ≤ 0.10). Dietary treatments were as follows: CON = basal diet; 3-NOP (3-nitrooxypropanol); TAN (*Acacia mearnsii*); and 3-NOP + TAN. Factor-level definitions: [3-NOP No] = CON + TAN; [3-NOP Yes] = 3-NOP + 3-NOP+TAN.

Previously, we showed that 3-NOP reduced CH_4_ by 22% in HF and 13% in BS, with little effect on lactational performance (Islam et al., 2025). Findings from the present study support and extend those observations by showing that breed-dependent differences in microbial community structure and function underlie divergent CH_4_ responses to 3-NOP, and may partly explain why HF show greater CH_4_ mitigation than BS. Interestingly, because both breeds were on the same basal diet, their communities shared the same core taxa, but differences in relative abundance were evident. Greater variability within BS masked clear breed separation, consistent with the lack of signal in unweighted UniFrac. Similarly, the absence of differences in weighted UniFrac indicated that the phylogenetic structure of dominant clades remained stable. In contrast, Bray–Curtis captured modest but consistent compositional shifts with 3-NOP, reflecting reweighting of shared taxa rather than lineage turnover. This pattern is in line with the known mechanism of 3-NOP: inhibition of methyl-coenzyme M reductase reduces CH_4_ while leaving the overall bacterial community largely intact, with targeted effects on H_2_-linked taxa (Romero-Perez et al., 2015; Pitta et al., 2022b). Such changes have been described as the microbial signature of 3-NOP supplementation (Pitta et al., 2022b). Breed differences in dominant bacterial genera aligned with previous reports of breed-dependent microbial structure (Paz et al., 2016; Gonzalez-Recio et al., 2018), supporting the idea that baseline ecology may condition responses to methanogenesis inhibitors. Despite these inherent differences, 3-NOP consistently enriched *Lachnospiraceae_NK3A20_group* in both breeds; other responses diverged: *Acetitomaculum* declined only in HF, whereas *Succinivibrionaceae_UCG-002* decreased in BS but increased in HF, suggesting that host genetics play a role in determining microbial profiles mediated via H_2_ fluxes.

At the functional level, within-breed correlation analyses showed that microbial-trait relationships were generally broader in HF and more limited in BS. In HF, genera such as *Succinivibrionaceae* and *Lachnospiraceae* were more often linked with propionate and tended to show negative associations with CH_4_ under 3-NOP, suggesting that these groups can redirect H_2_ toward propionate pathways (Pitta et al., 2022b). In BS, fewer consistent correlations were detected, and those that did appear were more often associated with acetate and butyrate, pathways that release H_2_ and therefore contribute less to CH_4_ reduction. Although the effects were modest, this pattern suggests that HF cows may have a greater ability to channel reducing equivalents into lower-CH_4_ sinks, whereas BS cows appear to maintain a fermentation profile that favors acetate and butyrate. Across both breeds, correlations with DMI and milk yield were weak and inconsistent, echoing earlier findings that short-term microbial interventions do not always translate directly into production responses. Still, the breed patterns align with the concept of H_2_ allocation phenotypes (Kaplan-Shabtai et al., 2021; Stepanchenko et al., 2023), which describe how microbial communities partition H_2_ among competing sinks when methanogenesis is suppressed. Although correlations do not imply causality, our data suggest that HF cows kept stronger links between propionate-associated genera (e.g., *Succinivibrionaceae_UCG-001*) and reduced CH_4_, whereas BS cows retained connections between acetate and butyrate-associated genera such as *Prevotella* or *Butyrivibrio*. These observations are consistent with our earlier report of a breed × 3-NOP interaction (Islam et al., 2025) and support the view that inherent host-microbiome differences shape how H_2_ is redirected when methanogenesis is inhibited.

Finally, some limitations should be noted. The relatively small sample size may have reduced the statistical power to detect subtle effects. Studies with a higher number of animals, ideally with direct measurements of H_2_ alongside gas emissions, are needed to confirm whether these associative patterns reflect true functional differences between breeds. Rumination bolus sampling provided a practical, noninvasive proxy for rumen microbiota, but some oral contamination cannot be ruled out. Exhaled VFA were measured as relative ion intensities and expressed as mol % rather than absolute concentrations, which limits quantitative interpretation. At the same time, both approaches offered important advantages: bolus sampling enabled repeated, animal-friendly sampling and exhalomics provided a novel, noninvasive window to monitor rumen function.
